# Prediction Models for Periprosthetic Joint Infection: A Systematic Review of Traditional and Machine Learning Approaches

**DOI:** 10.1016/j.mcpdig.2026.100392

**Published:** 2026-08-10

**Authors:** Farzad Pourghazi, Seyed Mohammad Amin Alavi, Fabio Borgonovo, Francesco Petri, Takahiro Matsuo, Matthew P. Abdel, Cody C. Wyles, Andrea Gori, Aaron J. Tande, Elie F. Berbari

**Affiliations:** a Division of Public Health, Infectious Diseases and Occupational Medicine, Department of Medicine, Mayo Clinic College of Medicine and Science, Mayo Clinic, Rochester, MN; b Department of Orthopedic Surgery, Mayo Clinic, Rochester, MN; c Faculty of Medicine, Ahvaz Jundishapur University of Medical Sciences, Ahvaz, Iran; d Department of Infectious Diseases, “Luigi Sacco” University Hospital, Regional Center for Infectious Diseases (CEREMI), Milan, Italy; e Department of Infectious Diseases, Infection Control, and Employee Health, The University of Texas MD Anderson Cancer Center, Houston, TX; f Department of Biomedical and Clinical Sciences, University of Milan, Milan, Italy; g Centre for Multidisciplinary Research in Health Science (MACH), University of Milan, Milan, Italy

## Abstract

**Objective:**

To systematically review the development, validation, and performance of traditional and machine learning (ML) prediction models for periprosthetic joint infection (PJI) risk, diagnosis, and clinical outcomes.

**Methods:**

We conducted a systematic review according to PRISMA guidelines to identify studies that developed or validated multivariable prediction models for PJI using traditional statistical or ML approaches. Searches were performed on December 10, 2025, across Ovid Embase, MEDLINE, Scopus, Web of Science, ClinicalTrials.gov, and CENTRAL.

**Results:**

Among 4565 identified records, 34 studies met inclusion criteria. Reported discrimination varied across model types and clinical end points. Among traditional risk-prediction models, 54.5% showed acceptable or good discrimination (0.7 ≤ area under the receiver-operating characteristic curve [AUROC] < 0.8), and 27.3% showed excellent discrimination (0.8 ≤ AUROC < 0.9). Traditional diagnostic and outcome prediction models showed good to outstanding discrimination. Among ML models, 50.0% of risk models showed acceptable or good discrimination (0.7 ≤ AUROC < 0.8), while 60.0% of diagnostic models reported AUROC of 0.9 or more. However, calibration reporting was inconsistent, particularly among ML studies, and external validation was limited. Model performance generally decreased in external validation cohorts compared with derivation cohorts. Substantial heterogeneity among included studies and model designs precluded direct crossmodel comparisons; therefore, differences in AUROC should not be interpreted as evidence of superiority between approaches.

**Conclusion:**

Prediction models for PJI demonstrate variable performance across risk prediction, diagnosis, and outcome prediction settings. However, substantial clinical and methodological heterogeneity, limited calibration reporting, and inadequate external validation restrict assessment of model reliability and generalizability. Current evidence does not support conclusions regarding the superiority between approaches.

Total joint arthroplasty (TJA) ranks among the most performed orthopedic procedures worldwide. Projections suggest that by 2030, the annual volume of primary total knee arthroplasty and total hip arthroplasty procedures in the United States will reach approximately 3.48 million and 572,000, respectively.^1^ Despite improvements in surgical techniques and perioperative care, periprosthetic joint infection (PJI) continues to affect approximately 2% of patients undergoing arthroplasty, a rate that has remained largely unchanged over time.^2^ The management of PJI imposes a substantial financial and health care burden. Average hospital costs are estimated at approximately $89,000 for hip PJI and $116,000 for knee PJI per episode, and revision for PJI after primary total hip arthroplasty is reported to cost more than 5 times as much as revision for noninfectious indications. Lifetime costs for hip PJI may approach $391,000.^3^^,^^4^ Over a patient’s lifetime, hip PJI alone can generate costs approaching $391,000.^5^

Previous studies have identified several risk factors for hip and knee PJI, including male sex, younger age, type II diabetes, class II obesity, hypertension, hypoalbuminemia, rheumatoid arthritis, posttraumatic osteoarthritis, prior intra-articular injections before total knee arthroplasty, previous steroid use, active tobacco use, prolonged operative time, and perioperative blood transfusion.^6^^,^^7^ Logistic regression has been widely used in multiple studies to develop predictive models for the risk of PJI following TJA, as well as for the diagnosis and outcome prediction of PJI.^8^, ^9^, ^10^, ^11^

In recent years, artificial intelligence (AI) has garnered substantial traction in health care, largely owing to its growing role in predictive modeling.^12^ Machine learning (ML) empowers clinicians and researchers to effectively evaluate extensive and intricate medical datasets, thereby improving the identification of patterns and risk factors and predicting outcomes. By enhancing analytical precision, these capabilities ultimately support improved clinical decision making and more effective patient care.^13^ Recent studies have examined the application of ML techniques in predicting PJI following hip and knee arthroplasty.^14^, ^15^, ^16^

Previously, Naufal et al^17^ systematically reviewed 13 studies, including 7 external validation studies, evaluating prediction models for PJI treatment outcomes, and highlighted several limitations of the reported models. More recently, Narine-Nagy et al^18^ identified 12 studies investigating the use of ML for predicting and diagnosing PJI, with most studies demonstrating good predictive performance.

Building on our previous systematic review examining the predictive performance and generalizability of traditional and AI-based models for surgical site infection after spinal surgery,^19^ the present review aimed to provide a broader synthesis across 3 clinically distinct end points (risk prediction, diagnosis, and outcomes), while descriptively summarizing reported model performance across regression-based and AI/ML-based approaches, calibration reporting, and external validation.

## Methods

The current systematic review was developed, implemented, and reported in accordance with the revised procedures of the Preferred Reporting Items for Systematic Reviews and Meta-Analyses (PRISMA) guidelines.^20^ This systematic review was not prospectively registered. An internal review protocol used to guide the conduct of the review is provided in the Supplemental Materials (available online at https://www.mcpdigitalhealth.org/) for transparency; however, this protocol was not independently registered before the review was conducted.

### Eligibility Criteria

We included all eligible study designs, including cohort studies, case series, and case-control studies without age limitations, which formulated models for PJI diagnosis identification, PJI post-TJA prediction, or associated clinical outcomes, using either AI-based or traditional statistical methodologies. In addition, we included only studies that reported measures of discrimination, such as the C-statistic or the area under the receiver-operating characteristic curve (AUROC). We did not limit the search strategy to any language. No restrictions were applied based on surgical setting, and studies including primary, revision, or mixed arthroplasty populations were eligible; this heterogeneity was considered during qualitative synthesis. Studies were excluded if they (a) featured nonhuman participants; (b) were not peer-reviewed or lacked full-text access; (c) were systematic reviews or meta-analyses; or (d) were nonprimary publications, such as book chapters or studies without patient-level data. Studies were also excluded if they failed to propose a multivariable prediction model, did not publish or allow extraction of discrimination measures, or did not model a PJI-specific end point.

### Information Sources and Search Strategies

We conducted a systematic literature search for studies focusing on prediction models for the diagnosis, risk, or PJI outcomes, using either traditional statistical methods or ML techniques. Search strategies were constructed using a combination of relevant keywords and standardized indexing terms. The complete search strategies are presented in the Supplemental Materials. Searches were conducted on December 10, 2025, across multiple databases and registries, including ClinicalTrials.gov (2000+), Ovid Central (1991+), Ovid Embase (1974+), Ovid MEDLINE (1946+ including Epub Ahead of Print, In-Process, and other nonindexed citations), Scopus (1823+), and Web of Science Core Collection (Science Citation Index Expanded 1975+ and Emerging Sources Citation Index 2015+). All retrieved records were imported into Covidence (Veritas Health Innovation), and duplicates were removed before screening.

### Study Selection

The screening of abstracts and full-text articles was conducted using Covidence systematic review software. Two reviewers independently assessed all records to ensure consistency. Abstract screening was performed independently by 2 reviewers (F.Pourghazi and S.M.A.A.), with disagreements resolved through consensus. Full-text screening was also conducted by the same 2 reviewers, and any discrepancies were resolved by consensus between the reviewers.

### Data Extraction

Data extraction was initially piloted in an Excel spreadsheet using 5 articles from each group (AI-based and traditional models). Two reviewers (F.Pourghazi and S.M.A.A.) evaluated the extraction form, and it was subsequently refined based on feedback from the research team. A third investigator with expertise in AI (F.B.) reviewed and further optimized the spreadsheet. Through this iterative process, the form was revised to clarify ambiguities and minimize discrepancies among reviewers. Once finalized, 2 independent reviewers (F.Pourghazi and S.M.A.A.) extracted relevant data from all included articles and recorded it in separate Excel spreadsheets.

Extracted variables included study design and setting; arthroplasty type; PJI definition; model type (traditional vs AI/ML); modeled end point category (risk, diagnosis, or outcome); candidate predictors and final predictors; and reported performance metrics, including discrimination, calibration, and validation approach (internal vs external). When a single study reported both traditional and AI/ML models, it was extracted and summarized in both corresponding sections. When a study reported both model development and validation, development and validation results were extracted separately. After study selection, eligible studies were grouped by model type (traditional statistical or AI/ML) and predicted end point (risk, diagnosis, or outcome). Studies reporting both model types were included in both groups. External validation studies were summarized separately, and development and validation results were extracted separately when both were reported. We extracted all relevant results for the predefined outcome domains, including discrimination, calibration, and validation. When a study reported multiple models, we recorded the AUROC of the best-performing model as presented by the authors. Development and validation results were extracted separately when both were available. In addition to outcome data, we extracted study design and setting, arthroplasty type, PJI definition, modeled end point category, model type, candidate and final predictors, and validation approach. When information was missing or unclear, it was recorded as not reported, and no assumptions were made beyond the data provided in the original publication.

For the purposes of this review, we classified models according to both modeling approach and modeled end point. Traditional statistical models were defined as models based primarily on conventional regression-based or score-based approaches, such as logistic regression, Cox regression, or nomogram-derived models. Artificial intelligence/ML-based models were defined as ML or other computational approaches designed to learn complex nonlinear relationships or interactions from data, including random forest, gradient boosting, support vector machines, neural networks, deep learning, and related methods. When a study reported both regression-based models and AI/ML models, each approach was extracted separately and summarized in the corresponding category. This classification was used as a practical reporting framework and should not be interpreted as meaning that these groups are completely separate or hierarchically arranged, since many AI/ML methods including commonly used approaches such as logistic regression belong to the broader field of statistical learning.^21^ The distinction in this review reflects differences in how studies labeled and presented their models, rather than any fundamental methodological separation. Modeled end points were classified as risk, diagnosis, or outcome. Risk models were defined as models developed to estimate the future probability of developing PJI, typically before or around the time of arthroplasty, for purposes such as preoperative counseling, perioperative risk stratification, and optimization. Diagnostic models were defined as models developed to estimate the probability that PJI is present at the time of clinical evaluation, typically in the setting of a painful arthroplasty or suspected infection. Outcome models were defined as models developed to predict prognosis after PJI had been diagnosed or treated, including outcomes such as debridement, antibiotics, and implant retention (DAIR) failure; recurrence; reinfection; or the need for suppressive antimicrobial therapy.

### Risk of Bias and Applicability Assessment

The risk of bias (ROB) and the applicability of prediction models were assessed using the Prediction Model Risk of Bias Assessment Tool (PROBAST),^22^ which includes 20 signaling questions across 4 key domains: participants, predictors, outcomes, and analysis. The PROBAST findings are marked such that low ROB is green, unclear ROB is yellow, and high ROB is red.^22^ Risk of bias and applicability were independently assessed by 2 reviewers (F.Pourghazi and S.M.A.A.) using the PROBAST tool. Interobserver agreement for the independent preconsensus risk-of-bias assessments was quantified using percentage agreement and Cohen κ. Discrepancies were resolved through discussion and consensus.

### Effect Size Estimation

Discrimination was assessed using the C-statistic (also referred to as the concordance statistic, C-index, or AUROC curve), which quantifies the ability of a model to distinguish individuals who experienced the outcome from those who did not.^17^ To facilitate narrative synthesis, AUROC values reported in AI/ML studies were interpreted as equivalent to C-statistics. No additional data conversions or imputation of missing summary statistics were performed; when information was missing or unclear, it was recorded as not reported. The C-statistic spans from 0.5 to 1.0, with elevated values indicating superior discriminative performance. Predefined thresholds were as follows: C-statistic ≥ 0.9, outstanding; 0.8 ≤ C-statistic < 0.9, excellent; 0.7 ≤ C-statistic < 0.8, good/acceptable; 0.6 ≤ C-statistic < 0.7, fair; and 0.5 ≤ C-statistic < 0.6, poor.^23^

Calibration is the degree to which a predictive model correctly predicts the likelihood of a certain event.^23^ Calibration may be evaluated by several ways, including the Hosmer–Lemeshow (H-L) test, Brier score, and calibration plots. The H-L test assesses model fit by contrasting observed and anticipated event probabilities, yielding a *P* value.^24^ A *P* value of less than .05 signifies potential inadequacy of the model fit, whereas a *P* value of more than .05 shows a lack of evidence for poor model fit.^24^ The Brier score delivers a thorough evaluation of a prediction model’s accuracy for binary or categorical outcomes, with values spanning from 0 to 1. A score of 0 signifies flawless prediction, indicating optimal model efficacy.^24^ A calibration plot is a graphical tool based on the H-L test that categorizes patients according to projected risk levels, offering a visual assessment of the congruence between the model’s predictions and actual results.^23^

### Statistical Analyses

A meta-analysis could not be performed because of substantial heterogeneity across the included studies, including differences in patient characteristics (eg, surgical indications, operative techniques, and risk factors for PJI), modeling methodologies, variables assessed, and PJI definitions. It should be noted that we explored whether sufficiently homogeneous subgroups existed for quantitative synthesis; however, substantial methodological and clinical heterogeneity prevented meaningful meta-analysis. Consequently, the data were synthesized narratively and summarized using descriptive statistics. Individual study results were summarized in tables by model type and predicted end point. Study selection was shown in a PRISMA flow diagram, and risk-of-bias results are presented in a Supplemental Table 1 (available online at https://www.mcpdigitalhealth.org/). Overall findings were summarized narratively using descriptive statistics. Accordingly, pooled effect estimates and quantitative comparisons with confidence intervals were not considered appropriate.

## Results

### Search Strategy and Bias Assessment

The initial search identified 4565 studies. After removing 2455 duplicates, 2110 citations underwent title and abstract screening, of which 2035 were excluded. Seventy-five full-text articles were sought for retrieval, and 18 were not retrieved. A total of 57 full-text articles were assessed for eligibility. Twenty-three studies were excluded for the following reasons: no prediction model proposed (n=15), area under the curve (AUC)/C-statistic not reported (n = 5), or modeled end point not specific for PJI (n=3). Ultimately, 34 studies met inclusion criteria and were included in the qualitative synthesis (Figure).^8^, ^9^, ^10^, ^11^^,^^14^, ^15^, ^16^^,^^25^, ^26^, ^27^, ^28^, ^29^, ^30^, ^31^, ^32^, ^33^, ^34^, ^35^, ^36^, ^37^, ^38^, ^39^, ^40^, ^41^, ^42^, ^43^, ^44^, ^45^, ^46^, ^47^, ^48^, ^49^, ^50^, ^51^

**Figure:**
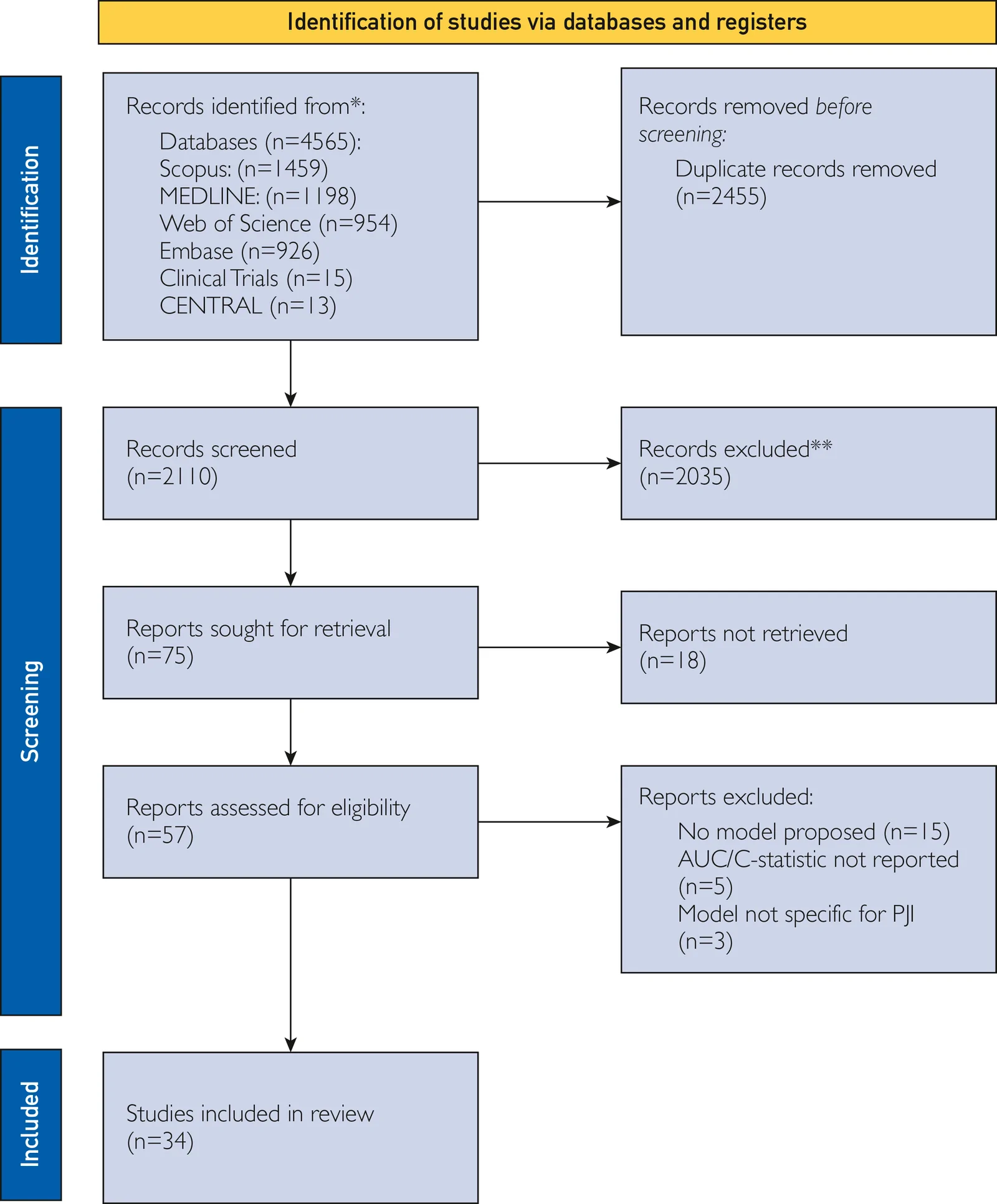
Preferred Reporting Items for Systematic Reviews and Meta-Analyses (PRISMA) flow chart of study selection. AUC, area under the curve; PJI, periprosthetic joint infection.

Using PROBAST, most studies were rated as having low ROB in the participants and predictors domains, while concerns were more frequently identified in the analysis domain (Supplemental Table 1). Several studies were classified as having a high ROB, primarily because of limitations in model development, handling of predictors, and incomplete reporting of validation procedures. Overall, applicability concerns were generally low across studies, with only a small number demonstrating moderate or high concerns in specific domains. Interobserver agreement for the independent preconsensus risk-of-bias assessments ranged from 79.4% to 100% across domains. Cohen κ values were 1.000 for participants, 0.737 for predictors, 0.773 for outcomes, 0.611 for analysis, and 0.702 for the overall risk-of-bias judgment. Across 170 paired domain-level judgments, 20 discrepancies occurred, most frequently in the analysis domain (7/34), followed by the overall risk-of-bias assessment (6/34). Eleven discrepancies involved low versus unclear ratings, 5 involved unclear versus high ratings, and 4 involved low versus high ratings. All discrepancies were resolved through discussion and consensus.

### Study Demographic Characteristics

Among the 34 included studies, both traditional statistical models and AI/ML-based models were represented. Eleven studies reported AI models^14^, ^15^, ^16^^,^^28^^,^^30^^,^^33^^,^^35^^,^^36^^,^^41^^,^^44^^,^^49^ and 15 reported traditional models,^8^, ^9^, ^10^, ^11^^,^^25^, ^26^, ^27^^,^^29^^,^^31^^,^^32^^,^^34^^,^^37^^,^^42^^,^^45^^,^^46^ two studies developed both AI and traditional models,^40^^,^^51^ and one study developed a traditional model and externally validated another study.^50^ Five studies externally validated other models. The general characteristics of the included studies are shown in Supplemental Table 2 (available online at https://www.mcpdigitalhealth.org/). The included studies addressed 3 main clinical decision points in PJI care. Risk-prediction models were intended to support preoperative counseling, risk stratification, and optimization before arthroplasty. Diagnostic models were designed to assist in the evaluation of a painful arthroplasty when infection is suspected. Outcome prediction models were aimed at informing postdiagnosis management decisions, including the likelihood of DAIR success, recurrence or treatment failure, and the need for longer-term suppressive antimicrobial strategies.

Two studies developed both traditional and AI/ML models and are therefore summarized in both sections.^40^^,^^51^ In addition, One study reported both model development and validation within the same publication,^50^ which were extracted and presented accordingly. It should be noted that, because of the heterogeneity among the included studies and their designs, any crossmodel comparison is methodologically unsupported. Therefore, differences in AUROC should not be interpreted as evidence of the superiority of one modeling approach over another. Grouped discrimination categories are presented for descriptive synthesis only and should not be interpreted as pooled estimates or direct quantitative comparisons across model types.

### Traditional Statistical Models

A total of 18 studies (Table 1) reported traditional statistical prediction models, spanning models for risk (n=11),^8^^,^^10^^,^^11^^,^^25^, ^26^, ^27^^,^^29^^,^^37^^,^^40^^,^^42^^,^^51^ diagnosis (n=3),^9^^,^^31^^,^^32^ and outcomes (n=4).^34^^,^^45^^,^^46^^,^^50^

**Table 1:** Characteristics of the Studies Using Traditional Prediction Models for PJI Stratified Based on Model End Point

Characteristic	Risk (n=11)	Diagnosis (n=3)	Outcome (n=4)
Study characteristics			
Retrospective studies	8 (72.7)	3 (100)	4 (100)
Prospective studies	1 (9.1)	0 (0)	0 (0)
Case-control studies	2 (18.2)	0 (0)	0 (0)
Single vs multicenter			
Single center	5 (45.5)	1 (33.3)	2 (50)
Multicenter	6 (54.5)	2 (66.7)	2 (50)
Study population size (total cohort)			
<500	0 (0)	3 (100)	2 (50.0)
≥500 to <1000	3 (27.3)	0 (0)	0 (0)
≥1000	8 (72.7)	0 (0)	2 (50)
NR	0 (0)	0 (0)	0 (0)
Case identification method			
Hospital record/registry	8 (72.7)	2 (66.7)	2 (50)
ICD coding	3 (27.3)	0 (0)	1 (25)
Other/NR	0 (0)	1 (33.3)	1 (25)
Arthroplasty characteristics			
Hip	3 (27.2)	1 (33.3)	2 (50)
Knee	0 (0)	0 (0)	0 (0)
Hip + knee	8 (72.8)	2 (66.6)	2 (50)
Primary vs revision arthroplasty			
Primary only	3 (27.3)	0 (0)	2 (50)
Revision only	1 (9.1)	2 (66.7)	0 (0)
Primary + revision	7 (63.6)	1 (33.3)	2 (50)
PJI definition used			
MSIS	8 (72.7)	2 (66.7)	1 (25)
ICM	0 (0)	1 (33.3)	1 (25)
Other/NR	3 (27.3)	0 (0)	2 (50)
Model type			
Multivariate logistic regression	8 (72.7)	2 (66.7)	4 (100)
LASSO	2 (18.2)	0 (0)	0 (0)
Nomogram	1 (9.1)	1 (33.3)	0 (0)
Candidate predictors included in final models^a^			
Age	5 (45.5)	0 (0)	0 (0)
Sex	8 (72.7)	0 (0)	1 (25)
Type of joint	5 (45.5)	0 (0)	1 (25)
Malnutrition	2 (18.2)	0 (0)	0 (0)
Obesity	10 (90.9)	0 (0)	0 (0)
BMI	10 (90.9)	1 (33.3)	2 (50)
Smoking or drinking status	2 (18.2)	0 (0)	1 (25)
Diabetes	7 (63.6)	1 (33.3)	0 (0)
Other comorbidities	9 (81.8)	1 (33.3)	3 (75)
Medications	3 (27.3)	0 (0)	0 (0)
Operative time	1 (9.1)	0 (0)	1 (25)
ASA physical status classification	5 (45.5)	0 (0)	3 (75)
Prior arthroplasty	7 (63.6)	0 (0)	1 (25)
Prior other operation	4 (36.4)	0 (0)	0 (0)
Type of surgery	NR	NR	2 (50)
Presence of sinus tract	NR	NR	1 (25)
Chronic renal failure	NR	NR	2 (50)
History of myocardial infarction	NR	NR	1 (25)
Liver cirrhosis	NR	NR	2 (50)
Use of general anesthesia	NR	NR	1 (25)
Laboratory biomarkers	3 (27.3)	2 (66)	3 (75)
Duration of symptoms	1 (9.1)	0 (0)	0 (0)
Previous PJI	3 (27.3)	0 (0)	0 (0)
High-risk wound at discharge	1 (9.1)	0 (0)	0 (0)
Time since index procedure	2 (18.2)	0 (0)	0 (0)
Synovial fluid WBC count	NR	1 (33.3)	1 (25)
Synovial fluid PMN percentage	NR	1 (33.3)	0 (0)
Erythrocyte sedimentation rate	NR	2 (66)	1 (25)
C-reactive protein	NR	2 (66)	2 (50)
Procalcitonin	NR	1 (33.3)	0 (0)
Platelet count	NR	1 (33.3)	0 (0)
Fever at the time of revision	0 (0)	1 (33.3)	0 (0)
Model performance reporting			
Best AUC/C-statistic ≥0.6 to <0.7	2 (18.2)	0 (0)	1 (25)
Best AUC/C-statistic ≥0.7 to <0.8	6 (54.5)	1 (33.3)	1 (25)
Best AUC/C-statistic ≥0.8 to <0.9	3 (27.3)	1 (33.3)	2 (50)
Best AUC/C-statistic ≥0.9	0 (0)	1 (33.3)	0 (0)
Calibration			
Calibration assessed	8 (72.7)	3 (100.0)	3 (75)
Calibration type^a^			
Quantitative calibration			
Calibration plots	7 (63.6)		2 (50)
Quantitative metrics calibration			
Hosmer–Lemeshow test	2 (18.2)	3 (100)	1 (25)
Brier score	1 (9.1)	—	—
Other	2 (18.2)	1 (33)	2 (50)
External validation			
External validation performed	6 (54.5)	1 (33.3)	1 (25)

Values are presented as n (%). NR means information not provided in the original study.

ASA, American Society of Anesthesiologists; AUC, area under the curve; BMI, body mass index; ICD, International Classification of Diseases; ICM, International Consensus Meeting; LASSO, least absolute shrinkage and selection operator; MSIS, Musculoskeletal Infection Society; NR, not reported; PJI, prosthetic joint infection; PMN, polymorphonuclear; WBC, white blood cell.

a Total can be >100% as >1 option could be selected.

### Traditional Models Predicting PJI Risk (n=11)

Risk-model studies were primarily retrospective (72.7%), with additional prospective (9.1%) and case-control designs (18.2%). Studies were commonly multicenter (54.5%), generally included cohorts of 1000 or more patients (72.7%), and most frequently identified cases using hospital records or registries (72.7%) or International Classification of Diseases coding (27.3%). Most studies included both hip and knee arthroplasties (72.8%) and most incorporated both primary and revision arthroplasties (63.6%). Further, PJI definitions varied, most frequently using Musculoskeletal Infection Society (MSIS) criteria (72.7%), but a notable proportion did not report a clear PJI definition (27.3%).^52^

Commonly evaluated candidate predictors included obesity and body mass index (90.9%), comorbidities (81.8%), sex (72.7%), prior arthroplasty (63.6%), diabetes (63.6%), and American Society of Anesthesiologists score (45.5%). No included study reported sex-stratified model performance or evaluated model discrimination or calibration separately by sex. Discrimination for best-performing models was most frequently in the good/acceptable range (C-statistic 0.7 to <0.8 in 54.5%), with fewer models achieving excellent (0.8 to <0.9 in 27.3%) discrimination. Calibration was reported in a minority of studies and varied in form, including both qualitative approaches (eg, calibration plots) and, less frequently, quantitative metrics such as the Brier score, calibration-in-the-large [CITL], or calibration slope. External validation was reported in 54.5% of studies.

### Traditional Models for Early PJI Diagnosis Identification (n=3)

All diagnosis-model studies were retrospective and predominantly multicenter (66.7%). These studies generally included smaller cohorts (<500 in 100%) and commonly focused on revision of arthroplasty populations (revision only in 66.7%). Further, MSIS criteria were the most frequently applied diagnostic definition (66.7%).^52^ Candidate predictors commonly included erythrocyte sedimentation rate and C-reactive protein (CRP) in 66.7%, procalcitonin, platelet count, fever, synovial fluid white blood cell, and polymorphonuclear in 33.3%.

Discrimination of best-performing diagnosis models ranged from good to outstanding (Table 1). All studies reported calibration. External validation was reported in 1 study (33.3%).

### Traditional Models Predicting PJI-Related Outcomes (n=4)

Outcome-model studies were all retrospective, and half of them were single-center and the other half were multicenter studies. Between single-center and multicenter designs. Cohort sizes were mixed (<500 in 50%; ≥1000 in 50%). These studies most included hip arthroplasty alone (50%) or combined hip and knee arthroplasty (50%). Definitions were heterogeneous, including MSIS,^52^ Centers for Disease Control and Prevention,^53^ and International Consensus Meeting.^54^ Commonly evaluated predictors included comorbidities (75%), American Society of Anesthesiologists physical status classification (75%), body mass index, chronic renal failure, and liver cirrhosis, and CRP in 50% of studies. Discrimination for best-performing outcome models was most frequently excellent (0.8 ≤ AUROC < 0.9 in 50%). Calibration was reported in 75% of studies, most commonly through qualitative approaches such as calibration plots (50%), whereas quantitative metrics (eg, Brier score, CITL, or calibration slope) were less frequently reported. External validation was reported in 25%.

### AI Models

A total of 13 studies reported AI/ML-based prediction models (Table 2), including models for risk (n=6),^14^, ^15^, ^16^^,^^40^^,^^49^^,^^51^ diagnosis (n=5),^28^^,^^30^^,^^33^^,^^36^^,^^41^ and outcomes (n=2).^35^^,^^44^ As individual AI studies may develop and report more than 1 AI model, the number of models presented in the tables exceeds the number of studies included. All AI/ML studies were retrospective.

**Table 2:** Characteristics of the Studies Using AI Models for PJI Stratified Based on Model End Point

Characteristic	Risk (n=6)	Diagnosis (n=5)	Outcome (n=2)
Study characteristics			
Retrospective studies	6 (100)	5 (100)	2 (100)
Prospective studies	0 (0)	0 (0)	0 (0)
Single vs multicenter			
Single center	5 (83.3)	4 (80)	1 (50)
Multicenter	1 (16.7)	1 (20)	1 (50)
Study population size (total cohort)			
<500	0 (0)	3 (60.0)	0 (0)
≥500 to <1000	0 (0)	0 (0)	1 (50)
≥1000	6 (100)	2 (40)	1 (50)
NR	0 (0)	0 (0)	0 (0)
Case identification method			
Hospital record/registry	3 (50)	1 (20)	1 (50)
ICD coding	1 (16.6)	1 (20)	0 (0)
Other/NR	2 (33.2)	3 (60)	1 (50)
Arthroplasty characteristics			
Hip	2 (33.3)	0 (0)	0 (0)
Knee	2 (33.3)	0 (0)	1 (100)
Hip + knee	2 (33.3)	5 (100)	1 (100)
Primary vs revision			
Primary	2 (33.3)	1 (20.0)	0 (0)
Revision	1 (16.6)	1 (20.0)	1 (50)
Primary + revision	2 (33.3)	3 (60.0)	1 (50)
Primary vs revision: NR	1 (16.6)	0 (0)	0 (0)
PJI definition used			
MSIS	4 (66.6)	2 (40.0)	2 (100.0)
ICM	2 (33.3)	3 (60.0)	0 (0.0)
Type of model^a^			
Gradient boosting (GBM/XGB/HGB)	5 (83.3)	3 (60)	0 (0)
Random forest	3 (50)	3 (60)	2 (100)
ANN	5 (83.3)	0 (0)	1 (50)
Support vector machine	3 (50)	1 (20)	0 (0)
K-nearest neighbors	2 (33.2)	1 (20)	0 (0)
Gaussian naïve Bayes	2 (33.3)	1 (20)	0 (0)
AdaBoost	1 (16.6)	0 (0)	0 (0)
Stochastic gradient descent	1 (16.6)	0 (0)	0 (0)
Ensemble ML model	0 (0)	1 (20)	0 (0)
Elastic net	0 (0)	1 (20)	1 (50)
Extra trees	0 (0)	1 (20)	0 (0)
Multilayer perceptron (or ANN)	0 (0)	1 (20)	0 (0)
Decision tree	0 (0)	1 (20)	0 (0)
Ensemble/stacking methods	0 (0)	2 (40)	0 (0)
Model development methods (AI/ML)			
Train/test split: 80/20	2 (33.2)	2 (40)	1 (50)
Train/test split: 70/30	1 (16.6)	1 (20)	0 (0)
Train/test split: Other reported	3 (50)	2 (40)	1 (50)
Crossvalidation			
Crossvalidation performed	5 (83.3)	4 (80)	2 (100)
Crossvalidation type			
5-fold crossvalidation	2 (33.2)	3 (60)	1 (50)
8-fold crossvalidation	0 (0)	0 (0)	1 (50)
10-fold crossvalidation	4 (66.6)	1 (20)	0 (0)
Candidate predictors included in final models^a^			
Age	4 (66.6)	2 (40)	2 (100)
Sex	4 (66.6)	0 (0)	1 (50.0)
Ethnicity	1 (16.6)	0 (0)	0 (0)
Type of joint	1 (16.6)	1 (20)	0 (0)
Malnutrition	1 (16.6)	0 (0)	0 (0)
Obesity	5 (83.3)	0 (0)	1 (50)
BMI	4 (66.6)	0 (0)	0 (0)
Smoking or drinking status	1 (16.6)	0 (0)	1 (50)
Diabetes	4 (66.6)	0 (0)	1 (50.0)
Other comorbidities	5 (83.3)	1 (20)	0 (0)
Medications	2 (33.2)	0 (0)	0 (0)
Operative time	1 (16.6)	0 (0)	0 (0)
ASA physical status classification	2 (33.2)	1 (20)	0 (0)
Laboratory biomarkers	4 (66.6)	3 (60)	1 (50)
History of septic arthritis	1 (16.6)	0 (0)	0 (0)
History of spinal anesthesia	1 (16.6)	0 (0)	0 (0)
Prior open surgery	2 (33.2)	0 (0)	0 (0)
Intravenous drug user	2 (33.2)	0 (0)	1 (50)
Depression	2 (33.2)	0 (0)	1 (50)
Type of insurance	2 (33.2)	0 (0)	1 (50)
Prior hospitalization	1 (16.6)	0 (0)	0 (0)
Time since index procedure	1 (16.6)	1 (20)	0 (0)
Wound exudation	NR	1 (20)	0 (0)
Sinus tract	NR	1 (20)	0 (0)
Increase of local skin temperature	NR	1 (20)	0 (0)
White blood cell count	NR	3 (60)	0 (0)
Blood urea nitrogen	NR	1 (20)	0 (0)
Creatinine	NR	1 (20)	0 (0)
Hemoglobulin	NR	2 (40)	0 (0)
C-reactive protein	NR	4 (80)	1 (50)
Erythrocyte sedimentation rate	NR	2 (40)	0 (0)
Platelet count	NR	3 (60)	0 (0)
Serum antithrombin	NR	1 (20)	0 (0)
Total serum protein	NR	2 (40)	0 (0)
Serum-related eosinophil	NR	1 (20)	0 (0)
α-Defensin	NR	1 (20)	0 (0)
Methicillin-resistant *Staphylococcus aureus* infection	NR	NR	2 (100)
Model performance reporting			
Best AUC/C-statistic ≥0.7 to <0.8	3 (50)	2 (40)	1 (50)
Best AUC/C-statistic ≥0.8 to <0.9	1 (16.6)	0 (0)	1 (50)
Best AUC/C-statistic ≥0.9	2 (33.3)	3 (60)	0 (0)
Calibration assessed	5 (83.3)	1 (20)	2 (100)
Calibration type^a^			
Quantitative calibration			
Calibration plots	4 (66.6)	1 (20)	2 (100)
Quantitative calibration			
Brier score	2 (33.2)	0 (0)	1 (50)
Other	3 (50)	0 (0)	0 (0)
External validation performed	0 (0)	1 (20)	1 (50)

Values are presented as n (%). NR means information not provided in the original study.

AI, artificial intelligence; ANN, artificial neural network; ASA, American Society of Anesthesiologists; AUC, area under the curve; BMI, body mass index; GBM, gradient boosting machine; HGB, histogram gradient boosting; ICM, International Consensus Meeting; ML, machine learning; MSIS, Musculoskeletal Infection Society; NR, not reported; PJI, prosthetic joint infection; XGB, extreme gradient boosting.

a Total can be >100% as >1 option could be selected.

### AI/ML Models Predicting PJI Risk (n=6)

All risk-model studies included cohorts of 1,000 or more patients; MSIS criteria were used in 66.6% of studies. Most common predictors were comorbidities in 83.3% and then age and sex in 66.6%. Discrimination was commonly good to excellent, with the best AUC values most frequently ranging from 0.7 to <0.8 (50.0%); 83.3% of studies reported calibration. Crossvalidation was reported in 83.3% (mostly 10-fold crossvalidation), whereas no study reported external validation.

### AI/ML Models for PJI Diagnosis (n=5)

Diagnosis-model studies were often conducted in smaller cohorts (<500 in 60.0%), frequently in revision arthroplasty populations, and commonly incorporated laboratory predictors (CRP, platelet, and white blood cell). Best AUC values were typically 0.9 or more (60.0%). Calibration was reported in 20.0%. External validation was reported at 20.0%.

### AI/ML Models Predicting PJI-Related Outcomes (n=2)

Outcome-model studies generally included larger cohorts and often modeled reinfection or treatment failure end points. One study reported best AUC values in the 0.7 to <0.8 and 0.8 to 0.9 range. Calibration and crossvalidation was reported for both studies.

### External Validation Studies

Six studies reported external validation results for previously developed models or for models that included both development and validation (Table 3). Validated models included kidney, liver, index surgery, cemented prosthesis, and C-reactive protein (KLIC),^45^ and other prediction models for PJI diagnosis, PJI risk, and PJI-related outcomes. Across these studies, external validation performance was generally lower than internal performance. For instance, 1 externally validated PJI diagnosis model demonstrated high internal discrimination with more modest external performance, while outcome models such as KLIC showed external AUC values in the acceptable range. Notably, 1 study both developed and externally validated a model within the same publication and was therefore represented in both the development and validation summaries.^50^ Overall, external validation was limited across both traditional and AI/ML models and was absent in several subgroups, particularly among AI/ML risk-prediction models.

**Table 3:** External Validation Studies of PJI Prediction Models

Reference, year	Validated model/score	Country	Study design	Endpoint	Total population	PJI cases	Sensitivity (%)	Specificity (%)	AUC/C-statistic	95% CI	Calibration assessed	Calibration method
Liukkonen et al,^38^ 2024	KLIC score^44^	Finland	Retrospective cohort	Outcome	283	283	44	82	0.63	0.55-0.72	No	NA
Löwik et al,^39^ 2018	KLIC score^44^	The Netherlands	Retrospective cohort	Outcome	386	386	52.2	70.9	0.64	0.59-0.69	No	NA
Sancho et al,^43^ 2022	Model by Shohat et al^44^	Spain	Retrospective cohort	Outcome	8639 arthroplasties screened 64 DAIR cases	64	82	44	0.69	NR	Yes	Hosmer–Lemeshow calibration plot
Yoon et al,^48^ 2024	Models by Tan et al,^11^ Del Toro et al,^29^ and Bülow et al^27^	The Netherlands, Portugal, and Spain	Retrospective cohort	Risk	2684	60	Tan: 72 Del Toro: 69 Bülow: 72	NR	Tan: 0.72 Del Toro: 0.69 Bülow: 0.72	Tan: 0.65-0.78 Del Toro: 0.59-0.78 Bülow: 0.62-0.81	Yes	Calibration plots, intercept, and slope
Zhang et al,^49^ 2023	Model by Berbari et al^25^ (Mayo)	USA	Retrospective cohort	Risk	405	10	83	79	0.88	0.85-0.91	Yes	Hosmer–Lemeshow
Slullitel et al,^51^ 2025	KLIC score^44^	Argentina	Retrospective cohort	Outcome	247	96	NR	NR	0.79	0.67-0.90	Yes	Calibration-in-the-large and calibration slope

AUC, area under the curve; DAIR, debridement, antibiotics, and implant retention; KLIC, kidney, liver, index surgery, cemented prosthesis, and C-reactive protein score; PJI, prosthetic joint infection.

## Discussion

In this systematic review of 34 studies evaluating prediction models for PJI, we found major differences across studies in patient populations, modeled end points (risk, diagnosis, and outcomes), predictor selection, and PJI definitions. These differences prevented quantitative pooling of model performance. Traditional statistical models (most often multivariable logistic regression) and AI/ML-based approaches (often tree-based ensemble methods such as random forest and gradient boosting) showed reasonable discrimination in many studies; however, the field remains limited by heterogeneity across study design and end points, inconsistent calibration reporting, and limited external validation. Across studies, the most frequent sources of bias were related to the domain analysis, including incomplete reporting of model development, inadequate handling of missing data, and limited external validation. Importantly, although we grouped models as traditional and AI/ML for reporting, these labels do not represent separate model families. Logistic regression, similar to many commonly used ML methods, is part of the broader statistical learning framework, and its classification as a traditional model reflects how studies labeled their methods rather than a true methodological distinction. Accordingly, small differences in AUROC between logistic regression and more complex AI/ML models should not be taken as evidence of superiority. Discrimination alone does not determine clinical usefulness; without adequate calibration, external validation, and demonstrated decision-making value, higher AUROC values rarely translate into meaningful real-world benefit. Therefore, differences in AUROC across studies should be interpreted cautiously and not viewed as evidence of clinically important advantage. Many AI/ML methods are part of statistical learning and share key concepts with regression-based approaches.^21^ Model performance also depends strongly on study design, case-mix, predictor availability, end point definitions, and, most importantly, validation strategy.^55^ For these reasons, differences in discrimination between traditional and AI/ML models across studies should be interpreted with caution and should not be taken as proof that one approach is better than the other. Overall, these findings show progress in PJI prediction modeling, but they also highlight the need for standard outcome definitions,^56^^,^^57^ clearer reporting of calibration, and more external validation to support generalizability and clinical use. Our findings are broadly consistent with recent systematic reviews reporting limited external validation and substantial heterogeneity among PJI prediction studies. However, the present review extends prior work by incorporating diagnostic and posttreatment outcome models alongside perioperative risk-prediction models.^17^^,^^18^

From a clinical perspective, discrimination alone is insufficient; models intended for patient counseling or management decisions must also provide reliable absolute risk estimates, clinically meaningful thresholds, and evidence of utility in practice.

Traditional statistical methods remain widely used for PJI prediction, especially for perioperative risk models and outcome models. Most traditional models used predictors that are available in routine practice and methods that are easier to interpret, including neutrophil to lymphocyte ratio.^58^ Many traditional models achieved acceptable to excellent discrimination, but performance varied across studies, likely because of differences in patient selection, case finding, follow-up, and PJI definitions. Calibration was reported more often in traditional models than in many AI/ML studies, which is important because clinicians need not only risk ranking but also reliable absolute risk estimates and clinically meaningful decision thresholds for counseling and management decisions. Still, even among traditional studies, calibration methods and reporting were often incomplete, which limits confidence in predicted probabilities when the model is applied in other settings (Table 1).

Artificial intelligence/ML models were increasingly common, especially for diagnostic endpoints and large retrospective datasets. Several AI/ML studies reported strong discrimination, which may reflect the ability of these methods to model complex relationships among predictors. However, many AI/ML studies relied mainly on internal validation (such as crossvalidation or a single train/test split), and few used external validations. This raises concern that some models performed well partly because they learned local data structures, documentation practices, or center-specific diagnostic workflows, rather than predictors that generalize well across institutions. In addition, AI/ML studies often provided limited details on key steps such as missing-data handling, class imbalance methods, feature engineering, and model tuning. This makes the models harder to reproduce and harder to compare fairly.

Although several AI/ML models showed high discrimination, these results should be interpreted cautiously. Contemporary reporting recommendations, including TRIPOD+AI, emphasize that transparent reporting of model development, preprocessing, validation, calibration, fairness, and reproducibility is essential before prediction models can be meaningfully compared or considered for implementation.^59^ In the present review, many included studies, particularly AI/ML studies, did not provide sufficient detail on key methodological steps, and only a minority underwent external validation. As a result, higher AUROC values may reflect local data structure or modeling flexibility or internal validation strategies rather than robust transportability or real-world clinical utility. Accordingly, the available literature should be interpreted less as evidence that AI/ML models are inherently superior to traditional approaches and more as a foundation for designing future PJI prediction models that are transparent, reproducible, externally validated, and clinically useful.

Across both modeling approaches, the main barrier to clinical use is generalizability. Many models were developed in single-center cohorts or selected populations, and only a minority were tested in independent external cohorts. Even when discrimination is strong in the derivation cohort, performance can drop in new settings because of differences in PJI prevalence, case-mix, perioperative practices, diagnostic thresholds, and follow-up. External validation is therefore essential before implementation. Validation studies should also report more than a single AUC/C-statistic and should consistently include calibration assessment. When calibration is poor, recalibration in the target population may be needed before clinical use. Notably, external validation was limited across the included studies and was absent in several important subgroups, particularly among AI/ML risk models.

Discrimination alone does not establish clinical usefulness. A model may separate high-risk and low-risk patients but still overestimate or underestimate absolute risk. In this review, calibration reporting was inconsistent, especially in AI/ML diagnostic studies, which limits interpretation of predicted probabilities. Few studies assessed clinical utility using decision-analytic methods such as decision-curve analysis or net benefit, although these methods are important for determining whether a model supports real clinical decisions. This is important in PJI because the harms of false-positive and false-negative results are different; overestimation may lead to unnecessary interventions, whereas underestimation may delay recognition or prevention of infection.

From a clinical perspective, discrimination alone is insufficient; models intended for patient counseling or management decisions must also demonstrate reliable calibration, external validity, clinically meaningful thresholds, and evidence of utility in practice. Risk-prediction models may inform preoperative counseling, optimization, and preventive planning before arthroplasty, but their performance is sensitive to how PJI is defined and to differences in local practice. Diagnostic models may support the evaluation of painful arthroplasty when infection is suspected, but their performance depends on the reference standard and on how consistently outcomes are labeled. Outcome prediction models may help guide treatment and prognosis after PJI has been diagnosed, such as decisions related to DAIR versus revision strategies, but these outcomes are influenced by surgical technique, organism factors, antimicrobial management, and follow-up duration. Although prediction models aim to estimate outcomes such as failure, recurrence, or functional recovery, clinical outcome studies following joint procedures have reported the complexity and heterogeneity of postoperative trajectories, further highlighting the need for robust and generalizable prediction models.^60^ For these models, clear end point definitions and external validation are especially important.

Future work should aim to move PJI prediction models from single-data set performance toward models that are reliable across settings. Clear definition of end points and use of standardized PJI criteria will improve benchmarking and may allow future quantitative synthesis. Reporting standards especially for AI/ML studies should be more consistent so that models can be reproduced and compared, including explicit reporting of missing-data handling, class imbalance methods, predictor processing, and the full validation approach. Model evaluation should also routinely include calibration metrics and, when possible, clinical utility analyses at clinically meaningful thresholds.

Some clinically relevant variables appear underused and may improve future models. Although many existing models rely heavily on general demographic and comorbidity variables, future risk models may benefit from incorporating more orthopedic-specific and perioperative predictors, including operative complexity, implant-related factors, antibiotic timing and redosing, transfusion-related factors, wound-related variables, and revision complexity. Host variables can be more accurately assessed using objective indicators, including glycemic control markers, dietary status, inflammatory markers, and pharmaceutical exposures that influence immune function. For diagnostic modeling, integrating synovial and serum biomarkers with microbiological characteristics and temporal trends in inflammatory markers may enhance utility, especially if models are constructed around specific decision points (eg, prerevision assessment vs early postoperative suspicion). For predicting outcomes, including details about the treatment strategy, the type of organism, resistance patterns, the length of time the antimicrobial used, the addition of rifampin, surgical factors, and early postoperative trends may help predict failure or recurrence. However, care must be taken in the design to avoid bias from postbaseline variables.

Finally, many models rely only on structured tabular data, so valuable information from imaging, pathology, operative reports, and other unstructured text may not be included. Multimodal approaches that integrate routine clinical variables with imaging features, pathology descriptors, microbiology time-series, and operative notes may better reflect real clinical complexity. However, these approaches must still meet the same standards for calibration, external validation, and transparent reporting. For clinical use, model outputs must be supported by proof of calibration within the target population and explicit details on the prediction methodology.

### Strengths and Limitations

This systematic review used a comprehensive search strategy, followed PRISMA, and assessed the ROB and applicability using PROBAST. Additionally, the ROB and applicability were assessed independently by 2 reviewers using the PROBAST tool, which enhances the reliability and transparency of the quality appraisal. We summarized findings by clinically meaningful endpoints, which helps connect model results to clinical decision points.

Several limitations in the evidence base should be emphasized. First, when studies reported multiple models, we extracted the best-performing model as presented by the original authors to maintain consistency across studies. In many studies, only the best-performing or final selected model was reported, whereas some AI/ML studies evaluated numerous candidate algorithms. Tabulating every candidate model was therefore not consistently feasible and would have substantially increased table length while potentially giving disproportionate weight to studies that tested many algorithms. However, this approach may introduce reporting bias and may overestimate overall model performance. Therefore, the findings should be interpreted as a summary of the best reported performance within each study rather than as a complete comparison of all candidate models. The included studies differed widely in design, patient selection, cohort construction, predictors, follow-up duration, and PJI definitions, which prevented meta-analysis and limited direct comparison across studies. Additionally, studies included varied in their patient populations, with some combining primary and revision arthroplasty cohorts, which may introduce clinical heterogeneity and limit the comparability of model performance across studies. External validation was not common, particularly for AI/ML models, and many studies relied only on internal validation, which can overestimate performance. Calibration was frequently unreported or inadequately assessed, with many studies relying on qualitative approaches such as visual calibration plots rather than reporting quantitative metrics (eg, Brier score, CITL, or calibration slope), thereby limiting confidence in estimated probabilities. Many studies, particularly those using AI/ML approaches, did not adequately report key modeling procedures, thereby limiting reproducibility and hindering fair comparison across models. None of the included studies reported sex-stratified model performance. This represents an important limitation in the evidence base and is not aligned with the Sex and Gender Equity in Research guidelines. Future studies should assess model discrimination, calibration, and clinical utility across sex to ensure equitable model performance. Finally, differences in case ascertainment and diagnostic workflow across centers may introduce center-specific bias and reduce transportability.

On the basis of the limitations identified in this review, future studies should aim to develop prediction models that are anchored to a clearly defined clinical use case, a standardized end point, and a prespecified prediction time horizon. For risk prediction, models should be intended for use before or around the time of arthroplasty and should rely on routinely available and potentially modifiable predictors to support preoperative counseling and optimization. For diagnostic models, models should be designed for patients undergoing evaluation of a painful arthroplasty and should incorporate clinically relevant laboratories, synovial, microbiologic, and other diagnostic variables using a consistent reference standard. For outcome prediction, models should address specific postdiagnosis management questions, such as the likelihood of DAIR failure, recurrence, reinfection, or the need for suppressive antimicrobial therapy, with clearly defined follow-up periods and outcome definitions. Regardless of whether the modeling approach is regression-based or AI/ML-based, future models should emphasize transparent predictor definitions, appropriate handling of missing data and class imbalance, reporting of both discrimination and calibration, and validation in independent external cohorts. Studies should also increasingly report clinical utility at decision-relevant thresholds and provide a clearer pathway for implementation in routine practice.

## Conclusion

In this systematic review of 34 studies, reported model discrimination varied across PJI risk, diagnostic, and outcome prediction models. However, the evidence base is limited by substantial heterogeneity, incomplete calibration reporting, and limited external validation, especially among AI/ML models. Their model performance depends strongly on cohort characteristics, end point definitions, and validation methods. Discrimination results should not be directly compared across studies as evidence that 1 approach is superior. Future research should use clear end points, report methods clearly, assess calibration and clinical utility, and test models in external cohorts to support the safe and wider use of PJI prediction models in clinical practice.

## Potential Competing Interests

Dr Abdel reports royalties from Stryker and OsteoRemedies; stock options in ForCast Orthopedics; royalties or other support from Springer; and board or committee appointments with AAHKS, IOEN, and The Knee Society, outside the submitted work. Dr Wyles reports personal consulting relationships with DePuy and Restor3D, outside the submitted work. All other authors report no competing interests.

## Declaration of Generative AI and AI-Assisted Technologies in the Writing Process

During the preparation of this work, the authors used generative AI to assist with language editing, readability, grammar, and typographical corrections. After using this tool, the authors reviewed and edited the content as needed and take full responsibility for the content of the publication.
