# Supplementary material for: Prediction Models for Periprosthetic Joint Infection: A Systematic Review of Traditional and Machine Learning Approaches
Source: Mayo Clin Proc Digit Health. 2026 Aug 10;4(4):100392. doi: 10.1016/j.mcpdig.2026.100392 (PMC13578976; doi:10.1016/j.mcpdig.2026.100392)
Supplement: Supplementary file 1 [file mmc1.docx]

Contents

[**Search Strategy:**	1](#_Toc235119891)

[**Supplementary Table 1.**	7](#_Toc235119892)

[**Supplementary Table 2.**	9](#_Toc235119893)

[**Prisma Checklist**	15](#_Toc235119894)

[**Review protocol**	18](#_Toc235119895)

[Review methods	19](#_Toc235119896)

# **Search Strategy:**

**Ovid MEDLINE(R) and In-Process, In-Data-Review & Other Non-Indexed Citations <1946 to December 16, 2025>**

1	Periprosthetic Joint Infection/ or Prosthesis-Related Infections/	16298

2	("periprosthetic joint infection*" or "prosthetic joint infection*" or PJI or "periprosthetic infection*" or "prosthetic infection*").ti,ab,kf.	10276

3	1 or 2	21444

4	Forecasting/ or Probability/	156141

5	(forecast* or predict* or prediction* or projection* or probability or (risk adj1 (score* or stratification or calculator* or model* or equation* or assess* or predict*))).ti,ab,kf.	2923013

6	4 or 5	3024575

7	exp Models, Statistical/ or "Sensitivity and Specificity"/ or ROC Curve/	897207

8	(model* or nomogra* or partin-table* or regression or likelihood or sensitivity or specificity or concordance-index or c-index or receiver-operating-characteristic* or ROC or HSROC or SROC).ti,ab,kf.	6576578

9	7 or 8	6905351

10	exp Algorithms/ or Automation/ or Information Technology/ or exp Informatics/	1048381

11	(algorithm* or algorhythm* or random-forest* or latent or structural-equation or machine-learning or artificial-learning or deep-learning or network-learning or artificial-intelligence or ambient-intelligence or computational-intelligence or machine-intelligence or neural-network* or probabilistic-network* or data-mining or big-data or computer-reasoning or computer-vision or automated-reasoning or heuristic* or MCDM or (multi* adj1 decision) or expert-system* or fuzzy or natural-language-processing or sentiment-analysis or vector or bayes* or k-nearest-neighbo?r or automat* or information-technolog* or informatics or analytics).ti,ab,kf. or ai.ti,kf.	1520185

12	10 or 11	2135329

13	9 or 12	8119974

14	3 and 6 and 13	1245

15	exp Animals/ not Humans/	5402076

16	14 not 15	1231

17	limit 16 to yr="2000 -Current"	1198

18	limit 16 to yr="2000 -Current"	1198

**Embase:**

Embase <1974 to 2025 December 16>

1	("periprosthetic joint infection*" or "prosthetic joint infection*" or PJI or "periprosthetic infection*" or "prosthetic infection*").ti,ab,kw.	11625

2	(forecast* or predict* or prediction* or projection* or probability or (risk adj1 (score* or stratification or calculator* or model* or equation* or assess* or predict*))).ti,ab,kw.	3968824

3	(model* or nomogra* or regression or likelihood or sensitivity or specificity or concordance-index or c-index or receiver-operating-characteristic* or ROC or HSROC or SROC or algorithm* or random-forest* or machine-learning or artificial-intelligence or deep-learning or neural-network* or data-mining or bayes* or analytics).ti,ab,kw.	8997388

4	1 and 2 and 3	944

5	(exp animal/ or exp nonhuman/) not exp human/	7877518

6	4 not 5	932

7	limit 6 to yr="2000 -Current"	926

**Scopus :1459**

TITLE-ABS-KEY (

"periprosthetic joint infection*" OR "prosthetic joint infection*" OR PJI

OR "prosthetic infection*" OR "periprosthetic infection*"

)

AND

TITLE-ABS-KEY (

forecast* OR predict* OR prediction* OR projection* OR probability

OR (risk W/1 (score* OR stratification OR calculator* OR model*

OR equation* OR assess* OR predict*))

)

AND

TITLE-ABS-KEY (

model* OR nomogra* OR regression OR likelihood

OR sensitivity OR specificity OR "concordance-index" OR "c-index"

OR "receiver-operating-characteristic*" OR ROC OR HSROC OR SROC

OR algorithm* OR "machine learning" OR "artificial intelligence"

OR "deep learning" OR "random forest" OR "neural network"

OR "data mining" OR bayes* OR analytics

)

AND PUBYEAR > 1999

**Web of Science (954 results from 1999)**

TS=("periprosthetic joint infection*" OR "prosthetic joint infection*" OR PJI

OR "periprosthetic infection*" OR "prosthetic infection*")

AND

TS=(forecast* OR predict* OR prediction* OR projection* OR probability

OR "risk score*" OR "risk stratification" OR "risk calculator*"

OR "risk model*" OR "risk equation*" OR "risk assessment"

OR "risk prediction")

AND

TS=(model* OR nomogra* OR regression OR likelihood

OR sensitivity OR specificity OR "c index" OR "concordance index"

OR ROC OR HSROC OR SROC

OR algorithm* OR "machine learning" OR "artificial intelligence"

OR "deep learning" OR "random forest" OR "neural network"

OR "data mining" OR bayes* OR analytics)**Cochrane (Central) : 13 Results 2000-2025**

("periprosthetic joint infection" OR "prosthetic joint infection" OR PJI)

AND

(forecast* OR predict* OR prediction* OR projection* OR probability)

AND

(model* OR nomogram* OR regression OR likelihood OR sensitivity OR specificity

OR "c-index" OR "receiver operating characteristic" OR ROC OR HSROC OR SROC

OR algorithm* OR "random forest" OR "machine learning" OR "deep learning"

OR "artificial intelligence" OR "neural network" OR "data mining"

OR heuristic* OR MCDM OR "expert system" OR fuzzy

OR "natural language processing" OR bayes* OR automat*

OR informatics OR analytics)

**EBM Reviews - Cochrane Central Register of Controlled Trials <November 2025>**

**13 Results**

EBM Reviews - Cochrane Central Register of Controlled Trials <November 2025>

1	("periprosthetic joint infection*" or "prosthetic joint infection*" or PJI or "periprosthetic infection*" or "prosthetic infection*").tw.	373

2	(forecast* or predict* or prediction* or projection* or probability or (risk adj1 (score* or stratification or calculator* or model* or equation* or assess* or predict*))).tw.	153118

3	(model* or nomogra* or regression or likelihood or sensitivity or specificity or concordance-index or c-index or receiver-operating-characteristic* or ROC or HSROC or SROC or algorithm* or algorhythm* or random-forest* or machine-learning or artificial-intelligence or deep-learning or neural-network* or data-mining or bayes* or analytics).tw.	299848

4	1 and 2 and 3	13

5	limit 4 to yr="2000 -Current"	13

---------------------------------------

**Clinicaltrials.gov : 14 Results**

("periprosthetic joint infection" OR "prosthetic joint infection" OR PJI)

AND

(predict OR model OR "machine learning" OR "artificial intelligence" OR "deep learning" OR algorithm)

**ICTRP: 8 Results**

(periprosthetic joint infection OR prosthetic joint infection OR PJI)

AND

(predict OR model OR "machine learning" OR "artificial intelligence" OR "deep learning" OR algorithm)

------------------

**Supplementary Table 1.** Risk of bias and applicability assessment.

|  | Risk of bias | | | | | Applicability | | | |
| --- | --- | --- | --- | --- | --- | --- | --- | --- | --- |
| Author, year | Participants | Predictors | Outcome | Analysis | Overall | Participants | Predictors | Outcome | Overall |
| Berbari 2012 | Low | Low | Low | Low | Low | Low | Low | Low | Low |
| Buller 2012 | Low | Low | Low | Low | Low | Low | Low | Low | Low |
| Tan 2018 | Low | Low | Unclear | Unclear | Unclear | Low | Low | Low | Low |
| Del Toro 2019 | Low | Low | Low | High | High | Low | Low | Low | Low |
| Bülow 2022 | Low | Low | Low | Low | Low | Low | Low | Low | Low |
| Perni 2024 | Low | Low | Unclear | High | High | Low | Low | Low | Low |
| Espíndola 2022 | Low | Low | Low | Low | Low | Low | Low | Low | Low |
| Lang, 2025 | High | High | High | High | High | High | High | High | High |
| Klemt 2021 | Low | Low | Low | Low | Low | Low | Low | Low | Low |
| Bingham 2018 | Low | Low | High | High | High | Low | Low | High | High |
| Fu 2024 | Low | Low | High | High | High | Low | Low | High | High |
| Jenny 2016 | Unclear | Unclear | Unclear | High | High | Low | Low | Low | Low |
| Venäläinen 2021 | Low | Low | Low | Low | Low | Low | Low | Low | Low |
| Zhang 2023 | Low | Low | Low | Low | Low | Low | Low | Low | Low |
| Tornero 2015 | Low | Low | Low | High | High | Low | Low | Low | Low |
| Kheir 2018 | Low | Unclear | Low | High | High | Low | Low | Low | Low |
| Shohat 2020 | Low | Low | Low | Low | Low | Low | Low | Low | Low |
| Sancho 2022 | Low | Low | Low | Low | Low | Low | Low | Low | Low |
| Löwik 2018/19 | Low | Low | Low | Low | Low | Low | Low | Low | Low |
| Liukkonen 2024 | Low | Low | Low | Low | Low | Low | Low | Low | Low |
| Slullitel 2025 | Low | Low | Low | Low | Low | Low | Low | Low | Low |
| Yoon 2024 | Low | Low | Low | Low | Low | Low | Low | Low | Low |
| Kuo 2022 | Unclear | Unclear | Unclear | Low | Unclear | Low | Low | Low | Low |
| Maradit Kremers 2023 | Low | Low | Low | Low | Low | Low | Low | Low | Low |
| Maradit Kremers 2025 | Low | Low | Low | Low | Low | Low | Low | Low | Low |
| Chong 2025 | Low | Low | Low | Low | Low | Low | Low | Low | Low |
| Dragosloveanu 2025 | Unclear | Unclear | Unclear | Unclear | Unclear | Low | Low | Low | Low |
| Klemt 2023 | Low | Low | Low | Low | Low | Low | Low | Low | Low |
| Salimy 2025 | Low | Low | Unclear | Low | Unclear | Low | Low | Low | Low |
| Chen 2024 | Low | Low | High | High | High | Low | Low | Low | Low |
| Di Matteo 2025 | Low | Unclear | Unclear | Low | Unclear | Low | Low | Low | Low |
| Kawakami 2023 | Low | Low | Low | Low | Low | Low | Low | Low | Low |
| Paranjape 2023 | Low | Low | High | High | High | Low | Low | Low | Low |
| Klemt 2022 | Low | Low | Low | Low | Low | Low | Low | Low | Low |

**Supplementary Table 2. General characteristics of included model-development studies.** a and b) These studies developed both AI and traditional models. C) This study developed a traditional model and externally validated KLIC score (Table 3). Abbreviation**:** ACS-NSQIP: American College of Surgeons National Surgical Quality Improvement Program; AI: Artificial Intelligence; APL: aseptic prosthetic loosening; BMI: Body Mass Index; CDC: Centers for Disease Control and Prevention; CPRD: Clinical Practice Research Datalink; FU: Follow-up; HES: Hospital Episode Statistics; ICM: International Consensus Meeting; Infectious Diseases Society of America; IQR: Interquartile Range; ML: Machine Learning; MSIS: Musculoskeletal Infection Society; NR: Not reported; ONS: Office for National Statistics; PHA: Partial Hip Arthroplasty; PKA: Partial Knee Arthroplasty; PJI: Prosthetic Joint Infection; SD: Standard Deviation; THA: Total Hip Arthroplasty; TJA: Total Joint Arthroplasty; TKA: Total Knee Arthroplasty; Y : Year

| **Author (Year)** | **Country** | **Study Design** | **Single vs Multicenter** | **Total Population** | **Final PJI Cases** | **Arthroplasty Type** | **Primary vs Revision** | **Inclusion Criteria** | **Exclusion Criteria** | **Mean Age (SD)** | **Female n (%)** | **Definition of PJI Used** | **Model Type** | **Endpoint** |
| --- | --- | --- | --- | --- | --- | --- | --- | --- | --- | --- | --- | --- | --- | --- |
| **Chen, 2024** | China | Retrospective cohort | Single center | 254 (199 for development and 55 for validation cohort) | 97 | THA, TKA | Both | Patients with post-TJA complications evaluated for PJI vs APL with complete “in-time” indicators | Bone cancer; Mega prosthesis; inflammatory arthropathy; incomplete indicators | 64.6 ± 12.9 | NR (0.63) | MSIS 2011 | AI/ML | Diagnosis |
| **Di Matteo, 2025** | Italy | Retrospective cohort | Single center | 1360 | 219 | THA, TKA | Revision only | Adults undergoing elective revision THA or TKA with complete preop clinical & lab data | Primary THA/TKA; age <18 y; missing predictive features | NR | 785 (0.57) | MSIS 2018 | AI/ML | Diagnosis |
| **Kawakami, 2023** | Japan | Retrospective cohort | Multi-center | 133 | 50 | THA, TKA | Primary and revision | PJI: patients undergoing secondary surgery for PJI; non-PJI: primary arthroplasty with ≥1-year FU and no infection | Missing laboratory data; septic shock with fatal outcome | 68.7 | NR | ICM 2013 | AI/ML | Diagnosis |
| **Kuo, 2022** | Taiwan | Retrospective cohort | Single center | 323 | 190 | THA, TKA | Both | Revision hip or knee arthroplasty patients with complete ICM feature data (2000–2019) | Neoplasm; Mega prosthesis; metal-on-metal implant; acute postoperative (<3 mo) or acute hematogenous PJI; missing key features | NR | NR | ICM 2018 | AI/ML | Diagnosis |
| **Paranjape, 2023** | USA | Retrospective cohort | Single center | 22766 | 3873 | Hip, Knee | Both | Patients with hip or knee arthroplasty undergoing synovial fluid testing for PJI | Samples with poor specimen integrity; missing biomarker data; non-hip/knee joints | 67 | NR (48.6) | Modified ICM 2018 | AI/ML | Diagnosis |
| **Klemt, 2022** | USA | Retrospective cohort | Single center | 618 | 165 | TKA | Revision only | Patients undergoing revision TKA for confirmed PJI with ≥3 year follow-up | Prior revision surgery; missing or incomplete data | 67.2 ± 10.9 | 311 (50.3) | MSIS 2011 | AI/ML | Outcome |
| **Shohat, 2020** | USA + Europe | Retrospective cohort | Multi-center | 1174 | 405 | THA, TKA | Both | Patients with early acute or late acute PJI treated with DAIR (THA/TKA), meeting MSIS criteria, ≥1-y FU | Did not meet MSIS criteria; missing key data; FU <1 y | 70.1 ± 12.0 | NR | MSIS 2011 | AI/ML | Outcome |
| **Chong, 2025** | Hong Kong (China) | Retrospective cohort | Single center | 3483 | 81 | TKA | Primary only | Patients undergoing primary TKA with complete operative records and ≥1-yr FU | Revision TKA; partial knee arthroplasty; incomplete data | 70.4 ± 10.0 | 2544 (0.73) | ICM 2013 (MSIS-based) | AI/ML | Risk |
| **Dragosloveanu, 2025** | Romania | Retrospective cohort | Single center | 27854 | 302 | THA, TKA | NR | Consecutive pts undergoing THA/TKA ; EMR records with required variables. | NR | 62.35±12.38 | NR | MSIS/IDSA-based diagnostic workup (version NR) | AI/ML | Risk |
| **Klemt, 2023** | USA | Retrospective cohort | Single center | 1432 | 208 | TKA | All Revision | Consecutive aseptic revision TKA procedures | Patients with prior revision surgeries, missing perioperative data, or < 2y FU | 66.7 (21.5) | 722 (50.4) | 1 | AI/ML | Risk |
| **Maradit Kremers 2023 ^a^** | USA | Retrospective cohort | Single center | 22350 | 283 | Hip | Both | Patients who underwent partial or THA | NR | NR | NR | 1 | AI/ML | Risk |
| **Maradit Kremers, 2025 ^b^** | USA | Retrospective cohort | Single center | 58574 | 1277 | Hip, Knee | Both | Primary and revision THA/TKA (2000–2019) | Revision for infection; missing key covariates | 67 ± 13 | 31944 (55) | MSIS 2011 | AI/ML | Risk |
| **Salimy, 2025** | USA | Retrospective cohort | Multi-center | 51053 | 252 | PHA only | Primary only | Adults ≥18 y undergoing primary THA in ACS-NSQIP (2013–2020) | Age <18; bilateral THA; missing data; in-hospital death | 65 (±11.2) | NR | ICM 2018 | AI/ML | Risk |
| **Bingham, 2018** | USA | Retrospective cohort | Single center | 182 | 91 | THA, TKA | Both | Adults undergoing evaluation for possible chronic PJI after THA/TKA with complete diagnostic workup | Acute PJI; incomplete diagnostic data | NR | NR | MSIS 2011 | Traditional | Diagnosis |
| **Fu, 2024** | China | Retrospective cohort | Multi-center | 170 | 79 | THA, TKA | Revision only | Patients ≥50 years undergoing revision arthroplasty for suspected CPJI or aseptic loosening | Acute PJI; malignancy; autoimmune disease; recent antibiotics; incomplete data | 72.2 ± 7.4 | NR | ICM 2018 | Traditional | Diagnosis |
| **Jenny, 2016** | France | Retrospective cohort | Multicenter | 104 | 43 | Hip (THA) | Revision only | Rev THA records; random selection per center; ≥1y FU. | NR | 70±12 | 50 (0.48) | MSIS 2011 | Traditional | Diagnosis |
| **Kheir, 2018** | USA | Retrospective cohort | Multi-center | 1438 | 1438 | Hip, Knee | Both | Patients undergoing surgical treatment for confirmed PJI with ≥1-year follow-up | Mega prosthesis; missing demographic or surgical data | 64.2 ± 12.0 | 710 (49.4) | ICM 2013 | Traditional | Outcome |
| **Slullitel, 2025 ^c^** | Argentina | Retrospective cohort | Single center | 247 | 96 | Hip | Primary | Patients > 18 y with hip arthroplasty diagnosed with acute PJI | Index surgery had been one- or two-stage revision surgery (n = 97), FU<2 y, bilateral PJI and incomplete record | 70.03 (15.07) | 48 (50) | EBJIS | Traditional | Outcome |
| **Tornero, 2015** | Spain | Retrospective cohort | Single center | 222 | 222 | Hip, Knee | Both | Early PJI (<90 d after arthroplasty; symptoms <21 d) treated with DAIR; hip or knee arthroplasty | NR | 71.6 ± 10.7 | 121 (54.5) | MSIS 2011 | Traditional | Outcome |
| **Venalainen, 2021** | Finland | Retrospective cohort | Multi-center | 25919 | 296 | Hip | Primary | Primary THA with complete registry data | Revision THA; missing key preoperative data; non-standard or non-elective procedures; period; non-revision-managed infections | NR | NR | No formal diagnostic | Traditional | Outcome |
| **Berbari, 2012** | USA | Case-Control | Single center | Baseline model: 617 (301 PJI cases, 316 controls) 1-month model: 574 (after exclusions) | 301 (baseline); 258 (1-mo model) | THA, TKA | Both | Adults undergoing primary or revision THA/TKA (2001–2006) | Missing key data; PJI <30 day or inadequate FU | NR | 150 (0.5) | MSIS 2011 | Traditional | Risk |
| **Buller, 2012** | USA | Retrospective cohort | Single center | 10411 | 309 | THA, TKA | Both | Patients with hip or knee PJI treated with DAIR and polyethylene exchange | Non-DAIR procedures; incomplete data | NR | NR | MSIS 2011 | Traditional | Risk |
| **Bulow, 2022** | Sweden (derivation); Denmark (validation) | Retrospective cohort | Multi-center (national registries) | 88830 | 2173 | THA | Primary only | Patients undergoing elective primary THA in national registries | Non-primary THA; incomplete data | NR | NR | Other (Registry-based) | Traditional | Risk |
| **Del Toro, 2019** | Spain | Prospective cohort | Multi-center | 3569 | 65 | THA, PHA, TKA, PKA | Both | Adults undergoing hip or knee arthroplasty | Death unrelated to PJI within 3 months; missing baseline or perioperative data | NR | NR (69) | MSIS 2011 | Traditional | Risk |
| **Espíndola,2022** | Spain, Italy, France, Germany, UK, Netherlands | Case-control | Multi-center | 516 | 130 | THA, TKA | Primary only | Adults undergoing primary hip or knee arthroplasty (2014–2016) | Revision arthroplasty; incomplete data; non–S. aureus PJI | NR | NR | MSIS 2011 | Traditional | Risk |
| **Klemt, 2021** | USA | Retrospective cohort | Single center | 1081 | 293 | THA, TKA | Revision only | Revision THA or TKA for confirmed PJI with ≥2-year follow-up | Non-PJI revision; follow-up <2 years | 67.2 ± 10.8 | 504 (46.6) | MSIS 2011 | Traditional | Risk |
| **Lang,2025** | China | Retrospective cohort | Multi-center | 589 | 87 | THA | Primary only | Age ≥18; primary THA; complete records; ≥1-y FU | Systemic inflammatory disease; malignancy; coagulation disorders; APL; prior revision THA | NR | 38 (43.7) | MSIS 2018 | Traditional | Risk |
| **Maradit Kremers, 2023 ^a^** | USA | Retrospective cohort | Single center | 22350 | 283 | Hip | Both | Patients who underwent partial or THA surgery | NR | NR | NR | MSIS 2011 | Traditional | Risk |
| **Maradit Kremers, 2025 ^b^** | USA | Retrospective cohort | Single center | 58574 | 1277 | Hip, Knee | Both | Primary and revision hip or knee arthroplasty (2000–2019) | Revision for infection; missing key covariates | 67 ± 13 | 31944 (55) | MSIS 2011 | Traditional | Risk |
| **Perni, 2024** | UK | Retrospective cohort | Multi-center | 223324 joints | 1249 | Hip, Knee | Both | Hip or knee arthroplasty (2007–2014); age ≥30; ≥6 months CPRD registration | <6 months CPRD history; incomplete linkage to HES/ONS | NR | NR | Other (ICD-10–based administrative definition) | Traditional | Risk |
| **Tan, 2018** | USA | Retrospective cohort | Multi-center | 27717 | 1035 | Hip, Knee | Both | Primary and revision THA/TKA (2000–2014) at a high-volume center | Mega prosthesis; missing surgical/demographic data | 64.1 ± 12.1 (derivation); 69.2 ± 12.5 (external) | NR | Other (ICD-9–based institutional definition) | Traditional | Risk |

# **Prisma Checklist**

| **Section and Topic** | **Item #** | **Checklist item** | **Location where item is reported** |
| --- | --- | --- | --- |
| **TITLE** | | |  |
| Title | 1 | Identify the report as a systematic review. | Title page (manuscript title) |
| **ABSTRACT** | | |  |
| Abstract | 2 | See the PRISMA 2020 for Abstracts checklist. | pp. 1–2 (Abstract) |
| **INTRODUCTION** | | |  |
| Rationale | 3 | Describe the rationale for the review in the context of existing knowledge. | pp. 5–6 (Introduction) |
| Objectives | 4 | Provide an explicit statement of the objective(s) or question(s) the review addresses. | p. 6 (Introduction, objective) |
| **METHODS** | | |  |
| Eligibility criteria | 5 | Specify the inclusion and exclusion criteria for the review and how studies were grouped for the syntheses. | pp. 7, 9–10 (Eligibility criteria and grouping for synthesis) |
| Information sources | 6 | Specify all databases, registers, websites, organisations, reference lists and other sources searched or consulted to identify studies. Specify the date when each source was last searched or consulted. | pp. 7–8 (Information sources; search date) |
| Search strategy | 7 | Present the full search strategies for all databases, registers and websites, including any filters and limits used. | p. 7 and Appendix (complete search strategies) |
| Selection process | 8 | Specify the methods used to decide whether a study met the inclusion criteria of the review, including how many reviewers screened each record and each report retrieved, whether they worked independently, and if applicable, details of automation tools used in the process. | p. 8 (Study selection) |
| Data collection process | 9 | Specify the methods used to collect data from reports, including how many reviewers collected data from each report, whether they worked independently, any processes for obtaining or confirming data from study investigators, and if applicable, details of automation tools used in the process. | pp. 8–9 (Data extraction) |
| Data items | 10a | List and define all outcomes for which data were sought. Specify whether all results that were compatible with each outcome domain in each study were sought (e.g. for all measures, time points, analyses), and if not, the methods used to decide which results to collect. | pp. 9–10 (Data items and endpoint definitions) |
|  | 10b | List and define all other variables for which data were sought (e.g. participant and intervention characteristics, funding sources). Describe any assumptions made about any missing or unclear information. | pp. 8–10 (Other extracted variables; missing/unclear information) |
| Study risk of bias assessment | 11 | Specify the methods used to assess risk of bias in the included studies, including details of the tool(s) used, how many reviewers assessed each study and whether they worked independently, and if applicable, details of automation tools used in the process. | p. 10 (Risk of bias and applicability assessment) |
| Effect measures | 12 | Specify for each outcome the effect measure(s) (e.g. risk ratio, mean difference) used in the synthesis or presentation of results. | p. 11 (Effect size estimation) |
| Synthesis methods | 13a | Describe the processes used to decide which studies were eligible for each synthesis (e.g. tabulating the study intervention characteristics and comparing against the planned groups for each synthesis (item #5)). | pp. 9–10 (Grouping by model type and endpoint) |
|  | 13b | Describe any methods required to prepare the data for presentation or synthesis, such as handling of missing summary statistics, or data conversions. | pp. 9, 11 (Data preparation; missing data and no conversions/imputation) |
|  | 13c | Describe any methods used to tabulate or visually display results of individual studies and syntheses. | p. 12 (Statistical analysis; tables, PRISMA diagram, supplementary ROB table) |
|  | 13d | Describe any methods used to synthesize results and provide a rationale for the choice(s). If meta-analysis was performed, describe the model(s), method(s) to identify the presence and extent of statistical heterogeneity, and software package(s) used. | pp. 11–12 (Narrative/descriptive synthesis; rationale for no meta-analysis) |
|  | 13e | Describe any methods used to explore possible causes of heterogeneity among study results (e.g. subgroup analysis, meta-regression). | pp. 11–12 (Heterogeneity assessed descriptively; no subgroup meta-analysis) |
|  | 13f | Describe any sensitivity analyses conducted to assess robustness of the synthesized results. | Not applicable; no sensitivity analyses were conducted. |
| Reporting bias assessment | 14 | Describe any methods used to assess risk of bias due to missing results in a synthesis (arising from reporting biases). | pp. 23–24 (Strengths and limitations; potential reporting bias from best-performing-model extraction) |
| Certainty assessment | 15 | Describe any methods used to assess certainty (or confidence) in the body of evidence for an outcome. | Not applicable; no formal certainty-of-evidence assessment was conducted. |
| **RESULTS** | | |  |
| Study selection | 16a | Describe the results of the search and selection process, from the number of records identified in the search to the number of studies included in the review, ideally using a flow diagram. | p. 12 and Figure 1 (PRISMA flow diagram) |
|  | 16b | Cite studies that might appear to meet the inclusion criteria, but which were excluded, and explain why they were excluded. | p. 12 and Figure 1 (reasons for full-text exclusions) |
| Study characteristics | 17 | Cite each included study and present its characteristics. | pp. 13–17; Tables 1–3 and Supplementary Table 2 |
| Risk of bias in studies | 18 | Present assessments of risk of bias for each included study. | pp. 12–13 and Supplementary Table 1 |
| Results of individual studies | 19 | For all outcomes, present, for each study: (a) summary statistics for each group (where appropriate) and (b) an effect estimate and its precision (e.g. confidence/credible interval), ideally using structured tables or plots. | Tables 1–3 (individual study/model performance results) |
| Results of syntheses | 20a | For each synthesis, briefly summarise the characteristics and risk of bias among contributing studies. | pp. 13–17; Tables 1–3 and Supplementary Tables 1–2 |
|  | 20b | Present results of all statistical syntheses conducted. If meta-analysis was done, present for each the summary estimate and its precision (e.g. confidence/credible interval) and measures of statistical heterogeneity. If comparing groups, describe the direction of the effect. | Not applicable; no meta-analysis or other statistical synthesis was performed. |
|  | 20c | Present results of all investigations of possible causes of heterogeneity among study results. | Not applicable; no formal investigation of causes of heterogeneity was performed. |
|  | 20d | Present results of all sensitivity analyses conducted to assess the robustness of the synthesized results. | Not applicable; no sensitivity analyses were conducted. |
| Reporting biases | 21 | Present assessments of risk of bias due to missing results (arising from reporting biases) for each synthesis assessed. | pp. 23–24 (potential reporting bias discussed as a review limitation) |
| Certainty of evidence | 22 | Present assessments of certainty (or confidence) in the body of evidence for each outcome assessed. | Not applicable; no formal certainty-of-evidence assessment was conducted. |
| **DISCUSSION** | | |  |
| Discussion | 23a | Provide a general interpretation of the results in the context of other evidence. | pp. 17–23 (Discussion) |
|  | 23b | Discuss any limitations of the evidence included in the review. | pp. 23–24 (Strengths and limitations: limitations of the evidence base) |
|  | 23c | Discuss any limitations of the review processes used. | pp. 23–24 (Strengths and limitations: limitations of review processes, including best-model extraction) |
|  | 23d | Discuss implications of the results for practice, policy, and future research. | pp. 18–25 (clinical implications and future research) |
| **OTHER INFORMATION** | | |  |
| Registration and protocol | 24a | Provide registration information for the review, including register name and registration number, or state that the review was not registered. | pp. 6–7 (review was not prospectively registered) |
|  | 24b | Indicate where the review protocol can be accessed, or state that a protocol was not prepared. | pp. 6–7 and p. 25 (internal protocol provided in supplementary materials) |
|  | 24c | Describe and explain any amendments to information provided at registration or in the protocol. | Not applicable; no registered protocol or registration amendments. |
| Support | 25 | Describe sources of financial or non-financial support for the review, and the role of the funders or sponsors in the review. | Funding/Support statement (title page) |
| Competing interests | 26 | Declare any competing interests of review authors. | Title page |
| Availability of data, code and other materials | 27 | Report which of the following are publicly available and where they can be found: template data collection forms; data extracted from included studies; data used for all analyses; analytic code; any other materials used in the review. | p. 25 (Data availability statement) |

*From:* Page MJ, McKenzie JE, Bossuyt PM, Boutron I, Hoffmann TC, Mulrow CD, et al. The PRISMA 2020 statement: an updated guideline for reporting systematic reviews. BMJ 2021;372:n71. doi: 10.1136/bmj.n71. This work is licensed under CC BY 4.0. To view a copy of this license, visit <https://creativecommons.org/licenses/by/4.0/>

# **Review protocol**

Review title and timescale

**1 Review title**
Predictive Models for Periprosthetic Joint Infection (PJI): A Systematic Review and Meta-Analysis Comparing Machine Learning and Conventional Statistical Approaches

**2 Original language title**
English

**3 Anticipated or actual start date**
November 2025

**4 Anticipated completion date**
April 2026

## Review methods

**5 Research question**
Primary objective: To compare the predictive accuracy and methodological quality of machine learning (ML) versus traditional (conventional statistical) models in predicting periprosthetic joint infection (PJI) after joint arthroplasty

**Specific Research Questions:**
1. What ML algorithms and traditional models have been applied for PJI risk prediction?
2. How do their predictive performances (AUC/C-statistic, sensitivity, specificity, calibration) compare
3. What are the common methodological strengths and limitations in each approach (sample size, validation, calibration, bias)
4. What clinical or surgical variables are most frequently identified as significant predictors across studies?

**6 Searches**
Databases: Medline (Ovid), Embase, Scopus, Web of Science, and ClinicalTrials.gov. Grey literature will not be included. No language or publication-year restrictions.

The search strategy will combine controlled vocabulary and free-text terms (e.g., “arthroplasty,” “periprosthetic joint infection,” “machine learning,” “artificial intelligence,” “predictive model,” “logistic regression,” “risk model”). The librarian will provide finalized search strings. Results will be imported into Covidence for deduplication and screening [3].

**7 Condition or domain being studied**

Prosthetic Joint Infection (PJI) is a tremendous burden for individual patients as well as the global health care. Joint replacement is a life enhancing procedure for millions of people (1). With increasing frequency of joint replacement surgery in recent years prevalence of PJI is expected to rise in upcoming years (2).

There has been a rise in studies utilizing machine learning and AI algorithms to predict patient outcomes, also several published articles reported controversial results for the clinical prediction models performance for prediction of PJI (3, 4).

This review will synthesize evidence comparing ML-based predictive models and traditional statistical models.

**8 Participants/population**
Studies including adult patients (≥18 years) undergoing any primary or revision arthroplasty in which a predictive model for PJI was developed, validated, or compared Exclusion: models predicting unrelated outcomes (e.g., mechanical failure) or non-prosthetic infections.

**9 Intervention(s), exposure(s)**
Use of machine learning or artificial intelligence methods for predicting PJI

**10 Comparator(s)/control**
Traditional (non-ML) predictive or statistical models for PJI prediction.

**11 Types of study to be included**
Inclusion:
- Model development, validation, or comparison studies using clinical or registry data.
- Prospective or retrospective cohorts.

Exclusion:
- Case reports, reviews, letters, animal or cadaveric studies.

**12 Primary outcome(s)**
Predictive performance of models (C-statistic/AUC-ROC).

**13 Secondary outcomes**
Calibration (Hosmer–Lemeshow, Brier score, calibration plot
Validation type (internal/external)
Model transparency and reporting quality (TRIPOD adherence)
Most frequently included predictors.

**14 Screening and data extraction (selection and coding)**
Two reviewers will independently screen titles/abstracts and full texts in Covidence Data extraction will be piloted on 5–10 studies and finalized iteratively. Extracted data: study design, sample size, algorithm type, input variables, discrimination metrics, calibration, validation, and country. Disagreements will be resolved by consensus or third reviewer.

**15 Risk of bias (quality) assessment**
Prediction model studies will be assessed using the PROBAST tool. Publication bias will be evaluated with funnel plots and Egger’s test if ≥10 studies.

**16 Strategy for data synthesis**
Quantitative synthesis (meta-analysis) will be conducted when sufficient homogeneity exists in performance metrics. Pooled AUCs for ML vs. conventional models will be calculated using random-effects models. Heterogeneity assessed via I² statistic and Q-test. Subgroup analyses will be based on model type, data source, joint type, and validation method [3]. Narrative synthesis will complement the quantitative results.

**References:**

1.	Tande Aaron J, Patel R. Prosthetic Joint Infection. Clinical Microbiology Reviews. 2014;27(2):302–45.

2.	Kurtz S, Ong K, Lau E, Mowat F, Halpern M. Projections of Primary and Revision Hip and Knee Arthroplasty in the United States from 2005 to 2030. JBJS. 2007;89(4).

3.	Naufal E, Shadbolt C, Wouthuyzen-Bakker M, Rele S, Sahebjada S, Thuraisingam S, et al. Clinical prediction models to guide treatment of periprosthetic joint infections: a systematic review and meta-analysis. (1532-2939 (Electronic)).

4.	Karlin EA, Lin CC, Meftah M, Slover JD, Schwarzkopf R. The Impact of Machine Learning on Total Joint Arthroplasty Patient Outcomes: A Systemic Review. (1532-8406 (Electronic)).
